# Indocyanine green fluorescence image processing techniques for breast cancer macroscopic demarcation

**DOI:** 10.1038/s41598-022-12504-x

**Published:** 2022-05-21

**Authors:** Maria Leiloglou, Martha S. Kedrzycki, Vadzim Chalau, Nicolas Chiarini, Paul T. R. Thiruchelvam, Dimitri J. Hadjiminas, Katy R. Hogben, Faiza Rashid, Rathi Ramakrishnan, Ara W. Darzi, Daniel R. Leff, Daniel S. Elson

**Affiliations:** 1 Hamlyn Centre, Institute of Global Health Innovation, Imperial College London, London, UK; 2 Department of Surgery and Cancer, Imperial College London, London, UK; 3 Department of Breast Surgery, Charing Cross Hospital, Imperial College Healthcare NHS Trust, London, UK; 4 Department of Histopathology, Charing Cross Hospital, Imperial College Healthcare NHS Trust, London, UK

**Keywords:** Applied optics, Medical imaging, Breast cancer, Cancer imaging, Biomedical engineering

## Abstract

Re-operation due to disease being inadvertently close to the resection margin is a major challenge in breast conserving surgery (BCS). Indocyanine green (ICG) fluorescence imaging could be used to visualize the tumor boundaries and help surgeons resect disease more efficiently. In this work, ICG fluorescence and color images were acquired with a custom-built camera system from 40 patients treated with BCS. Images were acquired from the tumor *in-sit*u, surgical cavity post-excision, freshly excised tumor and histopathology tumour grossing. Fluorescence image intensity and texture were used as individual or combined predictors in both logistic regression (LR) and support vector machine models to predict the tumor extent. ICG fluorescence spectra in formalin-fixed histopathology grossing tumor were acquired and analyzed. Our results showed that ICG remains in the tissue after formalin fixation. Therefore, tissue imaging could be validated in freshly excised and in formalin-fixed grossing tumor. The trained LR model with combined fluorescence intensity (pixel values) and texture (slope of power spectral density curve) identified the tumor’s extent in the grossing images with pixel-level resolution and sensitivity, specificity of 0.75 ± 0.3, 0.89 ± 0.2.This model was applied on tumor *in-situ* and surgical cavity (post-excision) images to predict tumor presence.

## Introduction

Breast cancer is the most commonly diagnosed cancer worldwide, affecting 1 in 8 women during their lifetime^1^. Definitive treatment often involves surgery, taking the form of either mastectomy or breast conserving surgery (BCS). In appropriate patients, BCS combined with radiotherapy provides equivalent cancer outcomes to mastectomy, but with lesser morbidity^2^. The premise of BCS is to remove just the tumor *en-bloc* with a rim of healthy tissue, leaving the remaining breast behind. Compared to mastectomy, BCS facilitates improved cosmetic outcome and quality of life, and reduces the psychological burden^3^.

However, the main risk of BCS is that of positive margins, whereby tumor encroaches upon the rim of tissue surrounding the resection. This risk affects on average 19% of women undergoing BCS^4^ in the UK. This is a substantial problem, as patients with positive margins require further surgical intervention and have more breast tissue removed in an attempt to leave the cavity tumor-free. Patients undergoing reoperation therefore suffer the risks of said intervention, including: impaired cosmesis, delays to neoadjuvant treatment (radio/chemotherapy), psychological stress, and hospital and economic burden.

Various technologies are being trialed to combat this prevalent issue. Preoperative imaging such as mammography, ultrasound, and MRI are all diagnostic, and imperative for therapeutic planning. However, preoperative planning is not without flaws, as the position of the patient changes during surgery, thus affecting breast position and tumor location. Therefore, intraoperative feedback is required.

In BCS cases where the tumor is small or impalpable, the gold-standard technique is wire-guided localization (WGL). During WGL, a wire is implanted into the core of the tumor using ultrasound or stereotactic guidance. There are multiple variations on this technique using either magnetic, radioactive, or radar seeds with comparable results to WGL^5^. However, all of these technologies only localize the tumor core, without providing information on disease extent nor invasiveness. Following excision, the specimen undergoes radiography, which confirms whether the localizing technology (if used) has been retrieved, and that the tumor has been resected on a macroscopic level. However, specimen radiography suffers from low sensitivity^6^ therefore histopathological analysis is required.

Histopathology is the gold standard to determine ground truth on the completeness of resection. However, conventional histopathological processing takes a few days due to complex preparation and staining routines. Immediate processing using fresh frozen section enables intraoperative histopathological feedback on margins, but demands a histopathologist on standby. Furthermore, it delays the procedure thus putting the patient at risk of prolonged anesthetic, as well as inconveniencing both the surgeons and anesthetists whilst they await for a result. However, this technique is not commonly used as it is prone to false negatives^7^ and not all hospitals are able to facilitate the additional staff required on standby.

Imaging, optical spectroscopy, bioimpedance or mass spectrometry techniques are currently being clinically trialed in view of addressing positive margins^8^. However, the majority are operator dependent, and only provide feedback on a limited area of the tumor. Of these, fluorescence guided surgery (FGS) is promising, as it provides real-time intraoperative macroscopic visualization of the targeted tissue (i.e. tumor), which is easily interpretable. In FGS, a fluorescent contrast agent (fluorophore) is externally administered to the patient, and accumulates in the tumor^9^. Upon the fluorophore’s accumulation, its fluorescence can be captured with an FGS camera system and be utilized to guide tumor resection in real-time.

The basic components of a typical FGS system are^10^: the fluorophore of interest, light source for fluorescence excitation, optical lenses for light-collection, camera with emission filter to register light within the spectral emission of said fluorophore, instrument control, image processing and display software, and a display monitor. Moreover, for wider adoption by the research community and clinical translation, the below system features are essential:

- Easily adaptable optical lenses and filtration for accommodation of a range of fluorescent agents^11^ and working distances/field of view (FOV)^10,12^
- Good sensitivity to a wide range of fluorescent agents;
- Real-time surgical guidance provided through color imaging of the surgical scene augmented with fluorescence-signal derived information;
- Intuitive software interface (with optional remote control for optimized ergonomics);
- Automatic dark frame acquisition for ambient light compensation;
- Correction of variable sample conditions (tissue optical properties, tumor depth, working distance, etc.) for standardized fluorescence signal extracted metrics^13^.

Among the currently Food and Drug Administration (FDA) approved fluorophores, indocyanine green (ICG), is the most widely accepted fluorophore due to its near-infrared (NIR) spectral properties, favorable penetration depth, and low toxicity^9^. Upon its intravenous administration, ICG binds to intravascular plasma proteins, and has a half-life of approximately 5 min before it is cleared by the liver^14^. Thus, fluorescence imaging upon systemic ICG administration (during the angiography phase) can visualize the vasculature. Additionally, due to its propensity to remain within any type of vasculature into which it is placed, it is approved for use in imaging both blood and lymphatic vessels. Furthermore, due to its excretion via the liver, it can also be used to identify the biliary tract^15,16^. Its use in cancer imaging is experimental^17^ as it relies on the tumor being detected via the enhanced permeability and retention (EPR) effect. According to the EPR effect, ICG leaks into tumor tissue via porous vasculature and remains in the extracellular space due to impaired lymphatic outflow^18^. Under the EPR hypothesis, delayed fluorescence imaging after earlier ICG administration (thus enabling the EPR phase) could visualize only the tumor-retained ICG since the rest would have been cleared from circulation^14^.

The aim of FGS is to display a live stream of color images of the surgical scene, augmented with a pseudo-color map, indicating the macroscopic extent of the tumor (a “tumor probability map”). Under the afore-mentioned EPR effect hypothesis for ICG, the fluorescence image brightness could be used to extract this tumor probability map. However, apart from the underlying ICG concentration, the fluorescence image brightness also depends on a number of variable experimental parameters including tissue optical properties and ambient light^13^. This dependence can lead to intra-image brightness variations that could be compensated with pixel value normalization through division with the image of the reflected light at the fluorescence emission bands^13^. Alternatively, as ICG fluorescence images can visualize vasculature, image texture metrics^19^ could also be used to identify the more chaotic tumor vascular architecture. Vascular texture is less dependent on experimental parameters such as tissue absorption provided that there is still detectable fluorescence. Therefore, texture metrics could potentially be advantageous over ICG concentration in their use as tumor indicators^19^.

With respect to the overlay of the retrieved tumor probability map, the dominant technique is the use of a “uniform color” map (i.e. green). The more opaque the green color is, the higher the probability that the tumor is present^20^. This overlay technique is preferred because it allows the fluorescence to be used for guidance, without significantly impeding the surgeon's color view of the operative field.

We have previously proved the feasibility of performing ICG imaging with sub-mm resolution during an *in-vivo* BCS pilot study (REC 18/LO/2018), whereby the quartet of fluorescence image texture metrics, namely, Euler number, fractal dimension, and slope and intercept of the power spectral density (PSD) curves, demonstrated potential for accurate tumor classification^21^. In the follow-up clinical study (REC 19/LO/0927) of forty BCS patients, we found that fluorescence image intensity can also be useful for tissue classification. Moreover, when imaging was performed in the angiography phase after ICG administration, the tumor to background ratio (mean pixel intensity of tumor region divided by mean pixel of healthy region) was 3 ± 1.74, significantly greater (*p* = 0.02) than when imaging was performed in the EPR phase (2.1 ± 0.92)^22^. This finding suggested that the EPR hypothesis may not be the sole tumor identification mechanism in ICG fluorescence imaging.

The work presented here aims to use the data from these forty BCS patients to:

1. Test the hypothesis that fluorescence image texture metrics could be more accurate as tumor classification model predictors when imaging is performed during the angiography phase rather than the EPR phase.
2. Investigate the combination of fluorescence image intensity and texture metrics as complementary tumor classification model predictors (“Hybrid model”).
3. Investigate whether the use of the tumor histopathology grossed specimens helps improve the FGS diagnostic accuracy.

To answer the above questions, the logistic regression (LR) and Support Vector Machine (SVM) models are herein employed for training and validation. The LR model has been suggested for tissue class prediction^20^, given a certain fluorescence intensity value. This model has been thus far used in visualization of rat orthotopic glioma model^23,24^, rat cranial nerve *ex-vivo*^25^ and mouse xenografts *in-vivo*^26^. We previously used the LR model with fluorescence texture metrics in freshly excised breast-specimens^21^ which yielded 83% mean accuracy. The SVM model has been previously used on breast specimen autofluorescence spectra, yielding 100% sensitivity and specificity^27^ and with polarization-resolved fluorescence spectra, yielding 90.5% sensitivity and 90.7% specificity^28^ for the 621–700 nm band.

## Methods

### Patients

Forty patients undergoing BCS were recruited to this clinical study. The study was approved by a UK Research Committee (REC 19/LO/0927), informed consent was obtained from all patients and all experiments were performed in accordance with the Integrated Research Application System (IRAS) approved protocol. Patients were divided into two cohorts, namely: a) the angiography cohort (n = 20, the first 10 from 07/2020 to 10/2020 and last 10 from 01/2021 to 02/2021 recruits), and b) the EPR cohort (n = 20, 20 recruits from 10/2020 to 12/2020). In the angiography cohort, the ICG was injected approximately 5 min before tumor resection, whereas, in the EPR cohort, the ICG was administered approximately 25 min before tumor resection. The objective of this division was to investigate whether timing has any effect on the fluorescence image TBR, which was published elsewhere^22^. In the herein work, the difference in the tissue classification accuracy between the two cohorts is investigated. This investigation is presented for the fluorescence intensity and texture metrics as stand-alone or combined tissue classification predictors.

### Set-up and Image acquisition

**Figure 1:**
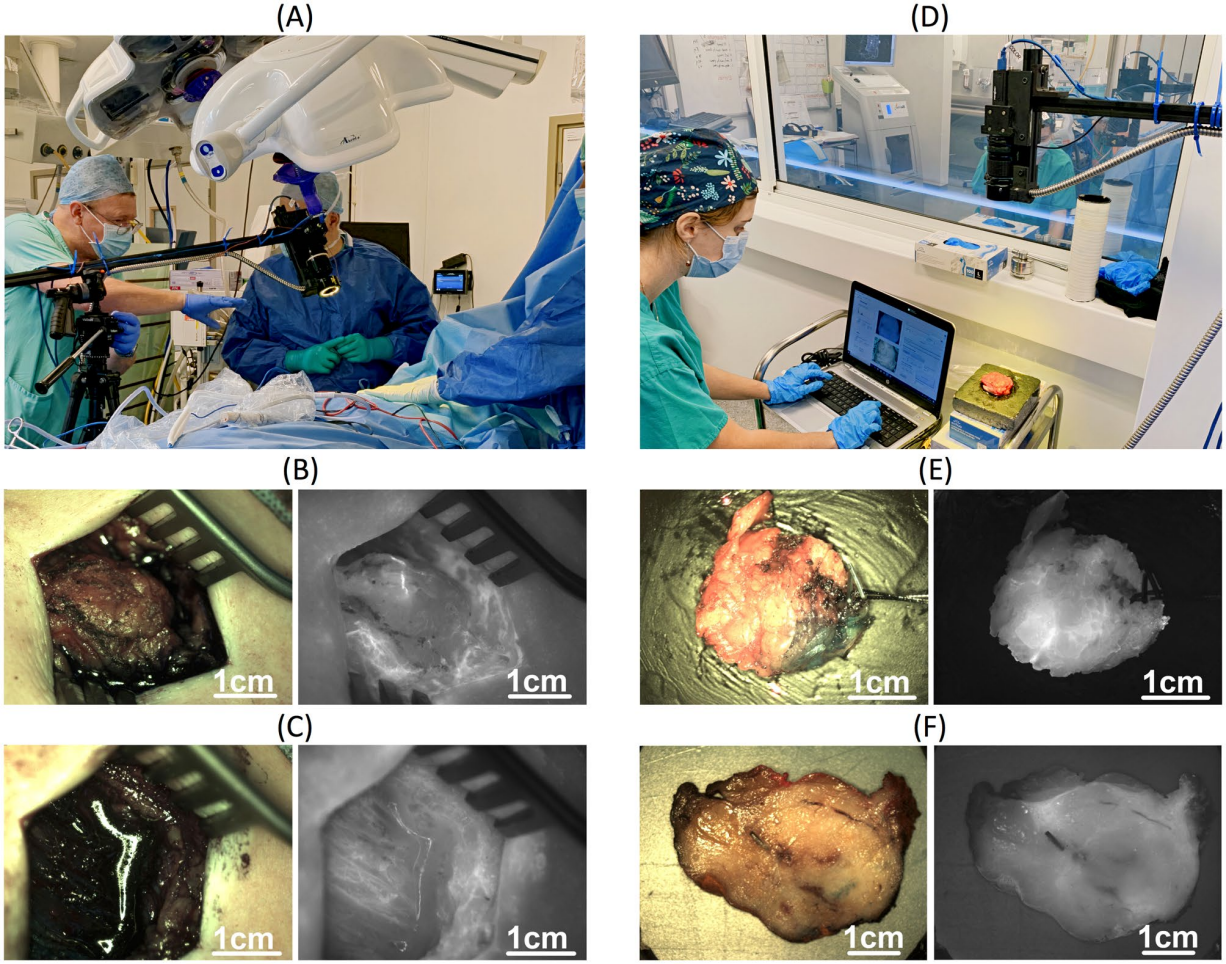
Demonstration of imaging with the in-house dual camera system and image category examples that have been used for either training/validation or prediction without subsequent validation of the classification models. (**A**) *In-vivo* imaging (as presented in^30^). (**B**) Tumor *in-situ* and (**C**) surgical cavity post-excision. These images were only used for trained model prediction (the trained models were applied to make predictions but could not be subsequently validated due to lack of ground truth). (**D**) *Ex-vivo* imaging. (**E**) Tumor *ex-vivo* and (**F**) tumor grossing in the histopathology lab, both used for model training and validation.

Upon systemic injection of 0.25 mg/kg ICG, an in-house fluorescence imaging system comprising two cameras, filtered light source, display monitor and controlling software^21^ (Fig. 1 and Figure S1) was used to acquire images. To capture the ICG fluorescence emission of breast tissue, a bandpass filter (825 nm central wavelength, 50 nm FWHM, Edmund Optics, Inc., Barrington NJ, USA) was used in front of the fluorescence camera. Moreover, to enable white light imaging and to eliminate overlap between excitation^21,29^ and emission spectra and autofluorescence a combination of a 500 nm long-pass and 750 nm short-pass filters was used for the illumination (TECHSPEC OD4, Edmund Optics, Inc., Barrington, NJ USA). The light source optical power (500–750 nm) at the light ring was ~ 0.8 W (measured via Coherent Field Max power meter with thermopile detector) with a light spot diameter of ~ 8 cm and average power density 4 mW cm^-2^. Color, fluorescence and dark frame (light source off) images were captured in the following settings, where the typical acquisition time for fluorescence images is given in parentheses:^21^.

1. the tumor *in-situ* prior to resection (Fig. 1B, 0.3–0.6 s);
2. the surgical cavity after resection (Fig. 1C, 0.3–0.6 s);
3. the tumor *ex-vivo* upon resection (specimen anterior and posterior side) (Fig. 1E, 0.8–1.0 s) and,
4. the tumor grossing in the histopathology (Fig. 1F, 0.3–0.8 s).

### Image treatment

**Figure 2:**
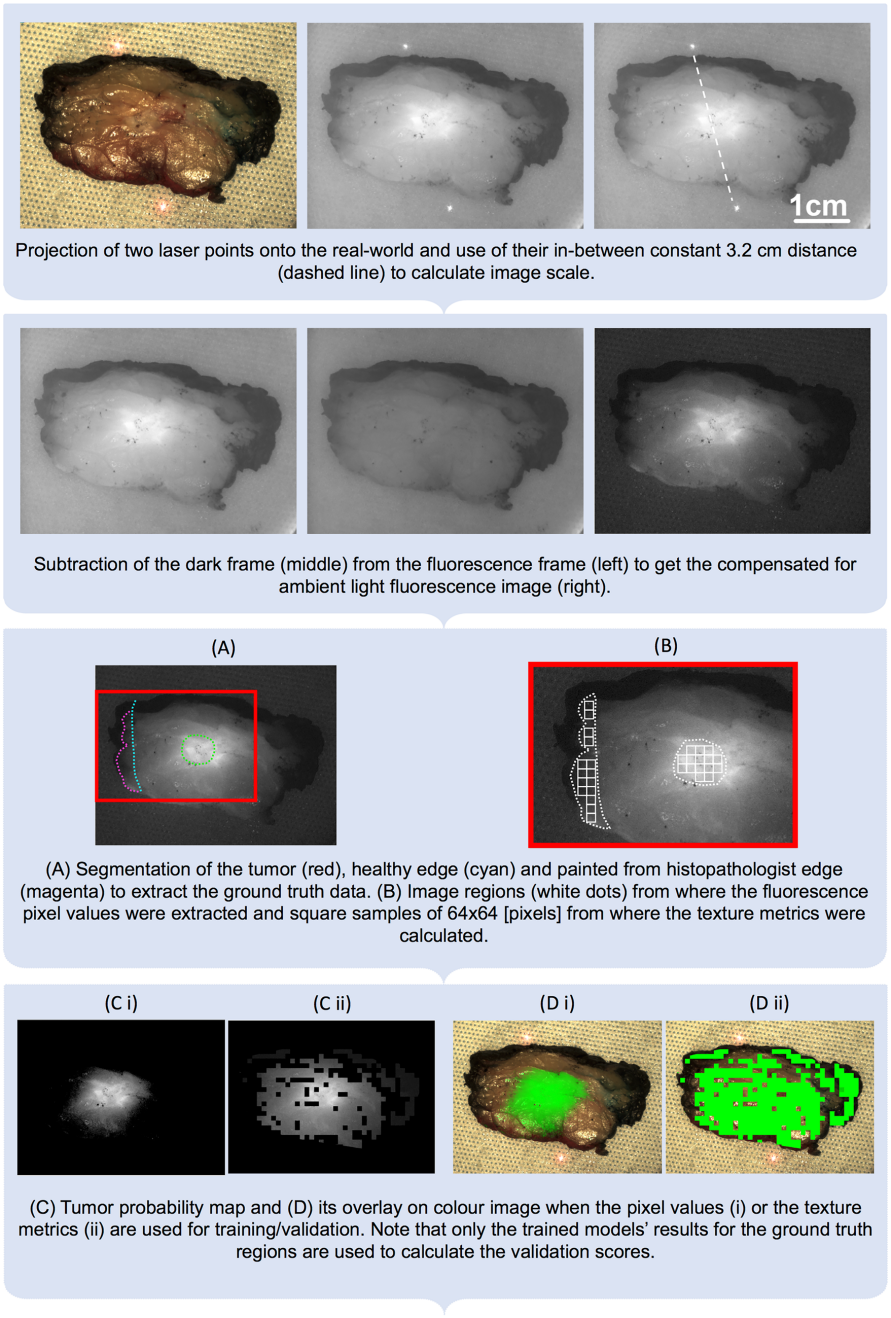
Image processing flow chart, demonstrating image scale extraction, dark frame subtraction, ground truth extraction and tumor probability map overlay of the fluorescence image pixel values and texture metrics separately. Areas which were true negatives were based on the histopathology report. True positives were taken from macroscopic identification of the tumor during grossing by a histopathologist. These areas were contoured by a clinical member of the team. In the tumor ex-vivo images, the specimen was oriented so that the fluorescence image was able to be compared with the corresponding radiography image. In this radiography image, the tumor core was indicated with a wire tip (WGL cases) and superior/lateral specimen views were shown with double and triple staples (demonstration in Fig. 3 of^21^). In the histopathology grossing data, specimen orientation was retrieved from the specimen facets inked to encode anterior/posterior, lateral/medial, or superior/inferior views.

To determine the size of each image, two parallel low power red laser (Class 1) beams were projected onto the scene (Fig. 2) at a constant 3.2 cm distance. Ground truth data (true normal and true tumor) was marked on tumor *ex-vivo* and grossed histopathology images, which was then used for training and validation. True negative margins were identified on a microscopic scale using histopathological reporting of the processed specimen. True positive margins were determined on a macroscopic scale. For the specimen images, ground truth was determined using specimen radiography images. For the grossed histopathology images, tumor sites were identified by a qualified histopathologist during grossing. Then, the images underwent segmentation of only the microscopic negative margins and macroscopically visible primary tumor. The exact location of the tumor could not be identified on the *in-vivo* images (tumour *in-situ* and surgical cavity images), therefore these images were missing the ground truth. Figure 2 provides an illustration of the detailed ground truth marking.

### Diagnostic model and predictors

To train and validate the logistic regression^31^ and SVM^32^ models, the tumor *ex-vivo* images (Fig. 1E) and in a separate analysis the histopathology grossing images (Fig. 1F) were used, as these were the two image types with marked ground truth. Initially, the fluorescence pixel values (intensity) of the ground truth tumor and healthy contours were used as separate predictors for the angiography and EPR cohorts. This analysis was implemented in a “case-wise” and in a leave-one-out cross validation (“LOOCV”) manner. In the case-wise approach, a random 70% of the ground truth data from each image was used to train, and the remaining 30% was used to validate the models. In the LOOCV approach the ground truth data from all the images except for one were used for training, and the remaining one was used for validation. In the LOOCV approach, pixel values were normalized within each image to the maximum intensity in order to account for inter-case variation (in illumination, contrast agent uptake and tumor depth). Validation was performed with the help of Receiver Operating Characteristic (ROC) curve analysis^33^ to extract sensitivity, specificity, and accuracy.

Afterwards, the texture metrics were validated as predictors, whereby the same ground truth regions were divided into square shaped samples of 64 × 64 pixels (Fig. 2), corresponding with a real-world dimension of approximately 2.5 × 2.5 mm. This low spatial resolution over which the texture metrics were extracted was chosen since it yielded the best validation scores in our previous work^21^. The quartet of texture metrics that we have previously identified as useful predictors^19^ was extracted from each square. Subsequently, those metrics were used as predictors for training and validation, following the same procedure as for the fluorescence intensity analysis.

The validation scores when the fluorescence pixel values or the texture metrics were used as predictors were finally compared between the angiography and the EPR cohort with the use of t-statistic^34^.

### Hybrid model

**Figure 3:**
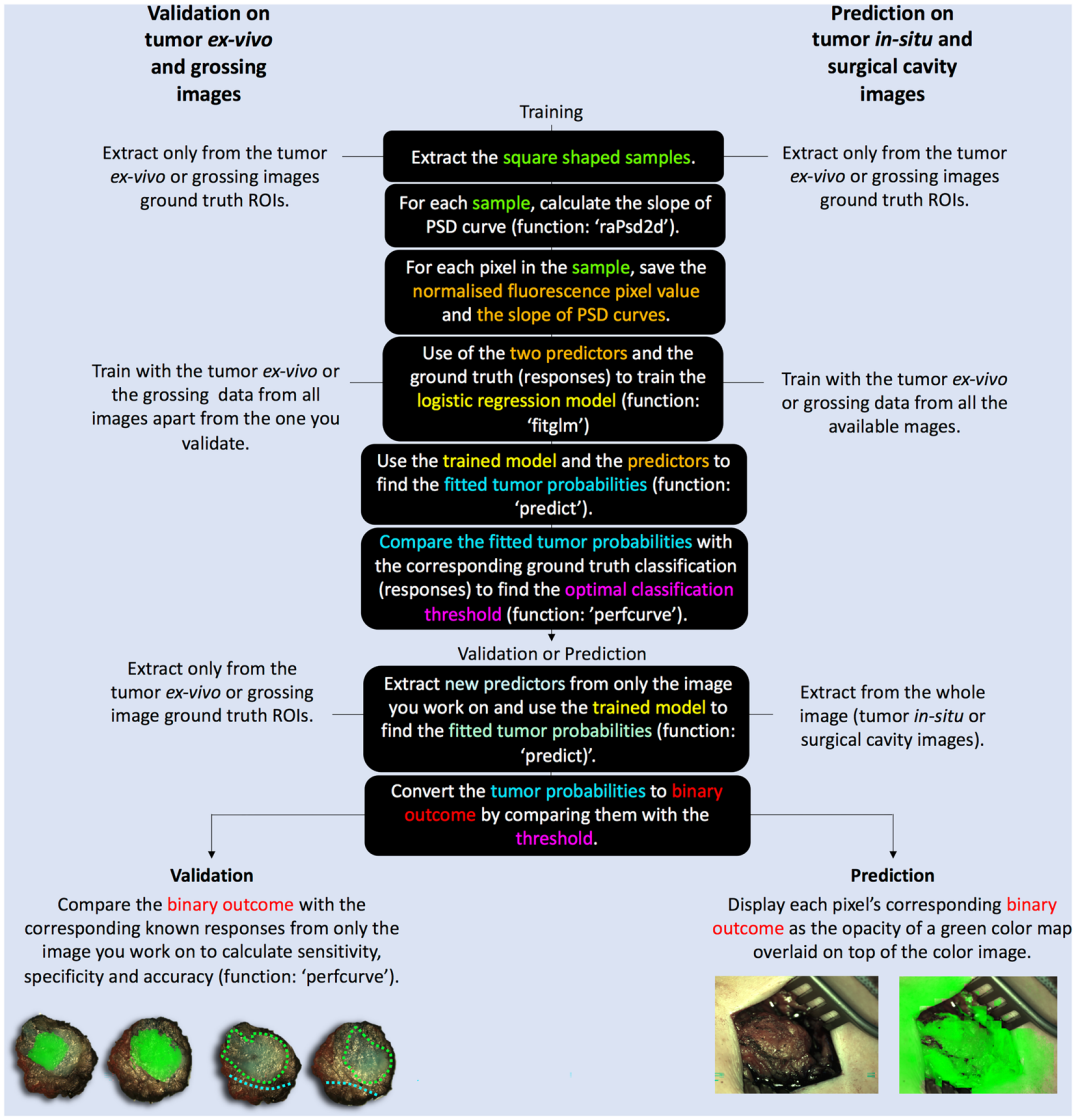
Hybrid model validation (with LOOCV approach) as well as prediction steps when both the normalized fluorescence pixel values and the slope of the PSD curves were used as predictors.

Combined use of two predictors was also applied in the model named thereafter as the “Hybrid model”. The aim of combining these two predictors was to obtain better sensitivity and specificity scores, compared to the scores achieved when each predictor was used alone. To accomplish this, in the hybrid model the combination of the predictors of fluorescence pixel value and slope of PSD curve was used to train and validate the logistic regression model in the LOOCV manner with the angiography cohort data. The choice of this combination is explained in the result and discussion sections. Finally, the hybrid model yielding the best validation scores (see Table 2) was trained anew with all the available ground truth data and applied to predict the tissue class (healthy/tumor) on the *in-vivo* images (tumor *in-situ* and surgical cavity) within the angiography cohort. Figure 3 presents the steps of the validation and application for predictions routines in the hybrid model.

### ICG laser induced fluorescence spectroscopy (LIFS) study

To estimate autofluorescence and ensure that the fluorescence signals in both the freshly excised *ex-vivo* tumor and formalin fixed histopathology grossing images originated from ICG, fluorescence spectra were acquired from five patients (four patients with ICG and one control).

After the administration of ICG as per the angiography cohort, fluorescence spectra acquisition was performed immediately after injection on the tumor *ex-vivo* samples. Spectra were collected using a 730 nm diode laser for ICG fluorescence excitation and spectrometer (FLAME, Ocean Optics Inc. Rochester, NY, USA) with in-built 750 nm long-pass emission filter (TECHSPEC® OD4, Edmund Optics Ltd, UK) for fluorescence acquisition. This combination was chosen to allow a comparison of the ICG emission spectra shape/ peak position between freshly excised and fixed in formalin samples. Excitation light (~ 5 mW at the fiber output) was delivered, and fluorescence was collected via a Y-shaped fiber-optic probe (LEONI Fiber Optics GmbH, Germany). Acquisition was performed by lightly touching the probe onto the surface of the tissue under the supervision of an experienced surgeon at points which were evenly distributed over the surface of the sample. Each tumor *ex-vivo* sample was positioned on a grid to ease sampled point location tracking. Thus, the approximative sampled position and corresponding classification of each point (healthy/cancer), according to the surgeon, was also recorded (if possible).

Overall, 625 spectra (50–250 spectra per sample) from tumors *ex-vivo* and 440 spectra (30–60 spectra × 10 slices for each sample) from histopathology grossing samples were acquired. As a control, 11 fluorescence spectra and 60 fluorescence images from a control sample (without ICG) were collected. Python 3.8.8 and SciPy package^35^ were used for data pre-processing, smoothing, filtering and maximum spectra estimation. Statistical analysis was performed using R^36^.

## Results

### Available data

Demographic/clinicopathological data and TBR analysis were presented in a separate publication^22^ and are provided as supplementary material (Supplementary Table S1). In brief, the two cohorts presented with similar demographics, with the majority having invasive ductal carcinoma with concomitant pre-invasive disease. 80 images of the tumor *ex-vivo* were obtained (anterior/posterior views), of which 50 were excluded due to the tumor lying deeper than 4 mm from the specimen surface. The excised specimen histopathology grossing images from 8 out of the 40 patient cases were also excluded due to unclear tumor location, remission, or due to technical malfunctions^22^.

### Validation with freshly excised ex-vivo tumor specimens

The validation scores (sensitivity, specificity, and accuracy) for the tumor *ex-vivo* specimen data are presented in Table 1. In this table, the results of logistic regression and SVM models are shown separately for the cases where the predictors were:

(i) only the fluorescence pixel values
(ii) only the slope of the PSD curves values or
(iii) both of the above values (hybrid model).

**Table 1:** The validation results of the tumor *ex-vivo* data analysis.

Whole tumor *ex-vivo* image analysis						
	Logistic regression			SVM model		
	Sensitivity	Specificity	Accuracy	Sensitivity	Specificity	Accuracy
**Case-wise analysis**						
*Fluorescence pixel values predictor*						
EPR	0.69 ± 0.3	0.95 ± 0.0	0.93 ± 0.1	0.64 ± 0.3	0.96 ± 0.0	0.80 ± 0.1
Angiography	0.80 ± 0.4	0.90 ± 0.1	0.92 ± 0.1	0.72 ± 0.4	0.94 ± 0.1	0.83 ± 0.2
*p* value	0.19	**0.02**	0.39	0.27	0.19	0.29
*Slope of PSD curves predictor*						
EPR	0.58 ± 0.5	0.86 ± 0.3	0.69 ± 0.3	0.42 ± 0.5	0.81 ± 0.4	0.61 ± 0.2
Angiography	0.74 ± 0.4	0.92 ± 0.2	0.81 ± 0.3	0.63 ± 0.5	0.63 ± 0.5	0.63 ± 0.5
*p* value	0.18	0.27	0.14	0.13	0.12	0.39
**LOOCV**						
*Fluorescence pixel values predictor*						
EPR	0.30 ± 0.3	0.96 ± 0.1	0.69 ± 0.1	0.31 ± 0.3	0.97 ± 0.0	0.69 ± 0.1
Angiography	0.60 ± 0.5*	0.85 ± 0.3*	0.81 ± 0.1*	0.58 ± 0.4	0.86 ± 0.3	0.82 ± 0.1
*p* value	**0.02**	0.07	** < 0.01**	**0.01**	** < 0.01**	**0.01**
*Slope of PSD curves predictor*						
EPR	0.64 ± 0.3	0.85 ± 0.3	0.84 ± 0.1	0.22 ± 0.4	0.78 ± 0.4	0.50 ± 0.0
Angiography	0.70 ± 0.4*	0.77 ± 0.3*	0.82 ± 0.2*	0.66 ± 0.5	0.61 ± 0.4	0.68 ± 0.2
*p* value	0.32	0.24	0.36	**0.01**	0.13	**0.01**

Fluorescence pixel values & slope of PSD curves predictor						
	Logistic regression (hybrid)					
	Sensitivity	Specificity	Accuracy			
Angiography	0.66 ± 0.5	0.74 ± 0.4	0.75 ± 0.2			

Results are presented for both the LG and SVM models and for both case-wise and LOOCV manner. The slope of PSD curves outperformed the rest of the texture metrics and therefore only this metric is presented here. Highlighted with asterisk scores of the LOOCV approach and angiography cohort show that the two predictors could complement each other to improve the model’s sensitivity and specificity. This is implemented in the hybrid model validation whose scores are presented in the last row of the table. The hybrid model was not implemented in the EPR cohort as in this cohort the sensitivity was inadequate (< 0.70) in both of the predictors. *p* values in bold indicate significant differences in validation scores at 5% significance level between the two cohorts.

**Figure 4:**
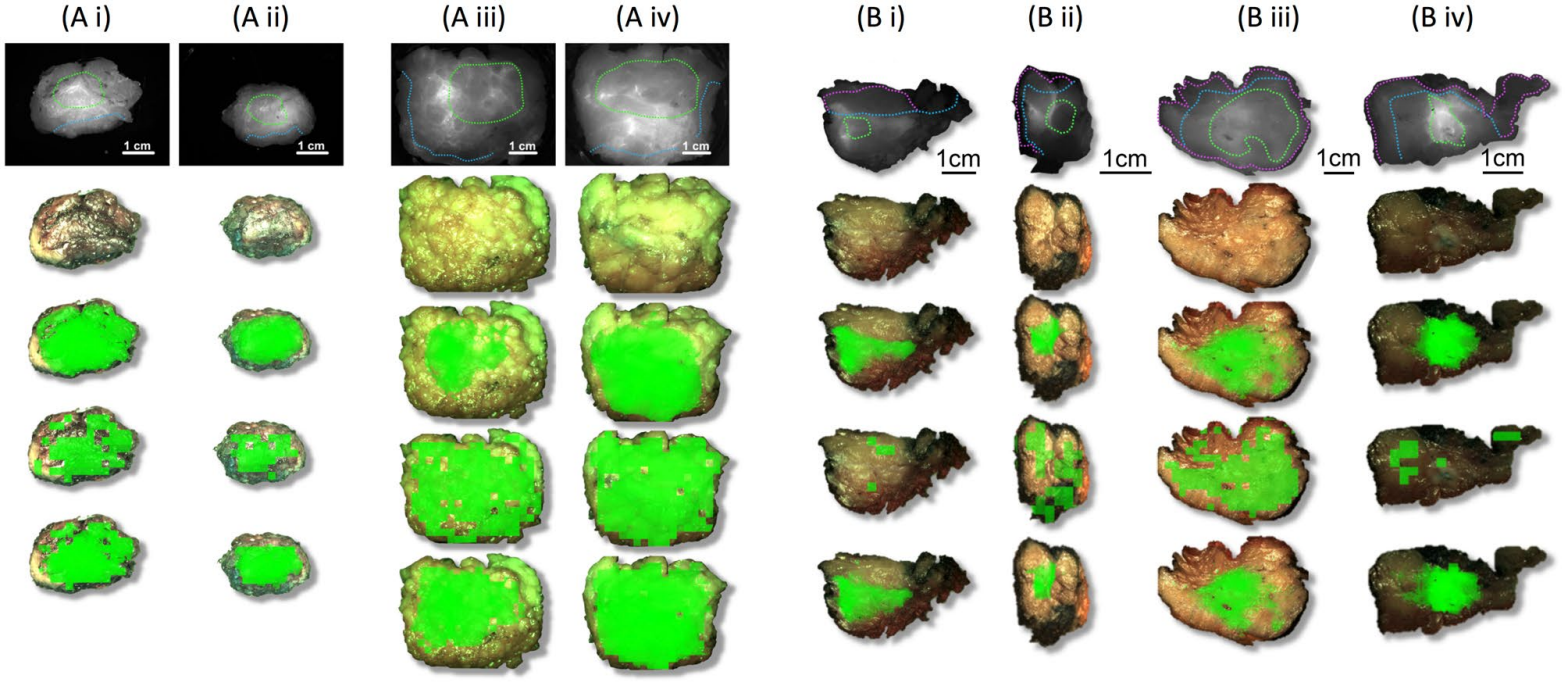
Overlay of the tumor probability maps on freshly excised tumor *ex-vivo* (**A**) and histopathology grossing (**B**) specimens from the angiography cohort. The whole *ex-vivo* tumor was imaged from two clinical cases (first: Ai-Aii and second: Aiii-Aiv) at the anterior (Ai, Aiii) and posterior (Aii, Aiv) views. Histopathology specimens are from four different clinical cases (Bi-Biv). First row: the raw fluorescence image marked for ground truth tumor (green contour) and healthy margin (blue contour). Second row: the corresponding raw color image. Third, fourth and fifth rows: overlay of the tumor probability maps on the color image when the logistic regression model was trained (in a LOOCV manner) with the angiography cohort *ex-vivo* tumor images (**A**) or the histopathology images (**B**) and the predictors were either only the normalized fluorescence pixel values (third row), or only the slope of the PSD curve values (fourth row) or the hybrid model (fifth row). Scale of each specimen (column) is indicated with a bar in the first row.

Examples of tumor probability map overlays on ex-vivo tumor images when the logistic regression model was trained in a LOOCV manner on fluorescent pixel values alone, on PSD curve slope alone, or on both (hybrid model) are provided in Fig. 4A.

### Validation with formalin-fixed histopathology grossing specimens

The validation scores for the histopathology grossing data are presented in Table 2. Overall, the best validation scores were those of the hybrid model when trained and validated on the angiography cohort histopathology grossing data: sensitivity: 0.75 ± 0.3, specificity: 0.89 ± 0.2 and accuracy: 0.84 ± 0.2. Examples of tumor probability map overlays on histopathology grossing images are presented in Fig. 4B.

**Table 2:** The validation results of the histopathology grossing data analysis, presented as with Table 1.

Histopathology grossing data analysis						
	Logistic regression			SVM model		
	Sensitivity	Specificity	Accuracy	Sensitivity	Specificity	Accuracy
**Case-wise analysis**						
*Fluorescence pixel values predictor*						
EPR	0.69 ± 0.3	0.92 ± 0.1	0.90 ± 0.1	0.60 ± 0.4	0.93 ± 0.1	0.77 ± 0.2
Angiography	0.82 ± 0.3	0.92 ± 0.1	0.93 ± 0.1	0.80 ± 0.3	0.95 ± 0.1	0.87 ± 0.2
*p* value	**0.02**	0.5	0.08	** < 0.01**	0.18	**0.01**
*Slope of PSD curves predictor*						
EPR	0.61 ± 0.4	0.93 ± 0.2	0.63 ± 0.3	0.36 ± 0.5	0.78 ± 0.4	0.57 ± 0.2
Angiography	0.61 ± 0.4	0.93 ± 0.1	0.72 ± 0.2	0.30 ± 0.3	0.90 ± 0.2	0.58 ± 0.1
*p* value	0.5	0.5	**0.05**	0.24	**0.04**	0.38
**LOOCV**						
*Fluorescence pixel values predictor*						
EPR	0.66 ± 0.3	0.91 ± 0.2	0.87 ± 0.2	0.36 ± 0.4	0.85 ± 0.3	0.61 ± 0.2
Angiography	0.82 ± 0.1*	0.72 ± 0.3*	0.86 ± 0.3*	0.70 ± 0.3	0.87 ± 0.3	0.82 ± 0.1
*p* value	** < 0.01**	**0.01**	0.43	0.07	0.4	**0.05**
*Slope of PSD curves predictor*						
EPR	0.58 ± 0.4	0.90 ± 0.2	0.64 ± 0.3	0.48 ± 0.4	0.52 ± 0.4	0.50 ± 0.0
Angiography	0.68 ± 0.3*	0.85 ± 0.2*	0.69 ± 0.3*	0.54 ± 0.4	0.73 ± 0.4	0.60 ± 0.2
*p* value	0.09	0.12	0.22	0.27	**0.01**	**0.01**

Fluorescence pixel values & slope of PSD curves predictor						
	Logistic regression (hybrid)					
	Sensitivity	Specificity	Accuracy			
Angiography	**0.75 ± 0.3**	**0.89 ± 0.2**	**0.84 ± 0.2**			

Once again, the scores highlighted with asterisks are complementary and show that the predictors have the potential to be combined. EPR cohort sensitivity scores were inadequate (< 0.70) thus the hybrid model was only implemented in the angiography cohort like in Table 1. The hybrid model scores highlighted in bold font in the last row are the best identified for LOOCV overall.* p* values in bold indicate significant differences in validation scores at 5% significance level between the two cohorts.

Overall, the angiography cohort had improved validation scores compared to the EPR cohort (*p* values in Tables 1, 2) when fluorescence pixel values were used as model predictors in agreement with our previous work^22^. This was also the case when the slope of PSD curves was used as predictor. Although not statistically significant, the following trends were observed: (a) the logistic regression outperformed the SVM model for both cohorts and (b) the case-wise analysis achieved better validation metrics than the LOOCV for the fluorescent pixel value analysis.

The sensitivity score (0.82) was higher when the fluorescence pixel values were the predictors, than when the PSD curve slope values were the predictors (0.68). On the contrary, the specificity score was higher with the PSD curve slope values as predictors (0.85) than with the pixel values as predictors (0.72). This was only the case for the logistic regression model/angiography cohort/ LOOCV combination (corresponding sections highlighted with asterisk in Tables 1 and 2) Therefore, both of the predictors (fluorescence pixel values and slope of PSD curves) were then used to train and validate the logistic regression model (the hybrid model) to investigate whether their combination could yield a good score for both sensitivity and specificity.

### Application of the hybrid model on the *in-situ* images

**Figure 5:**
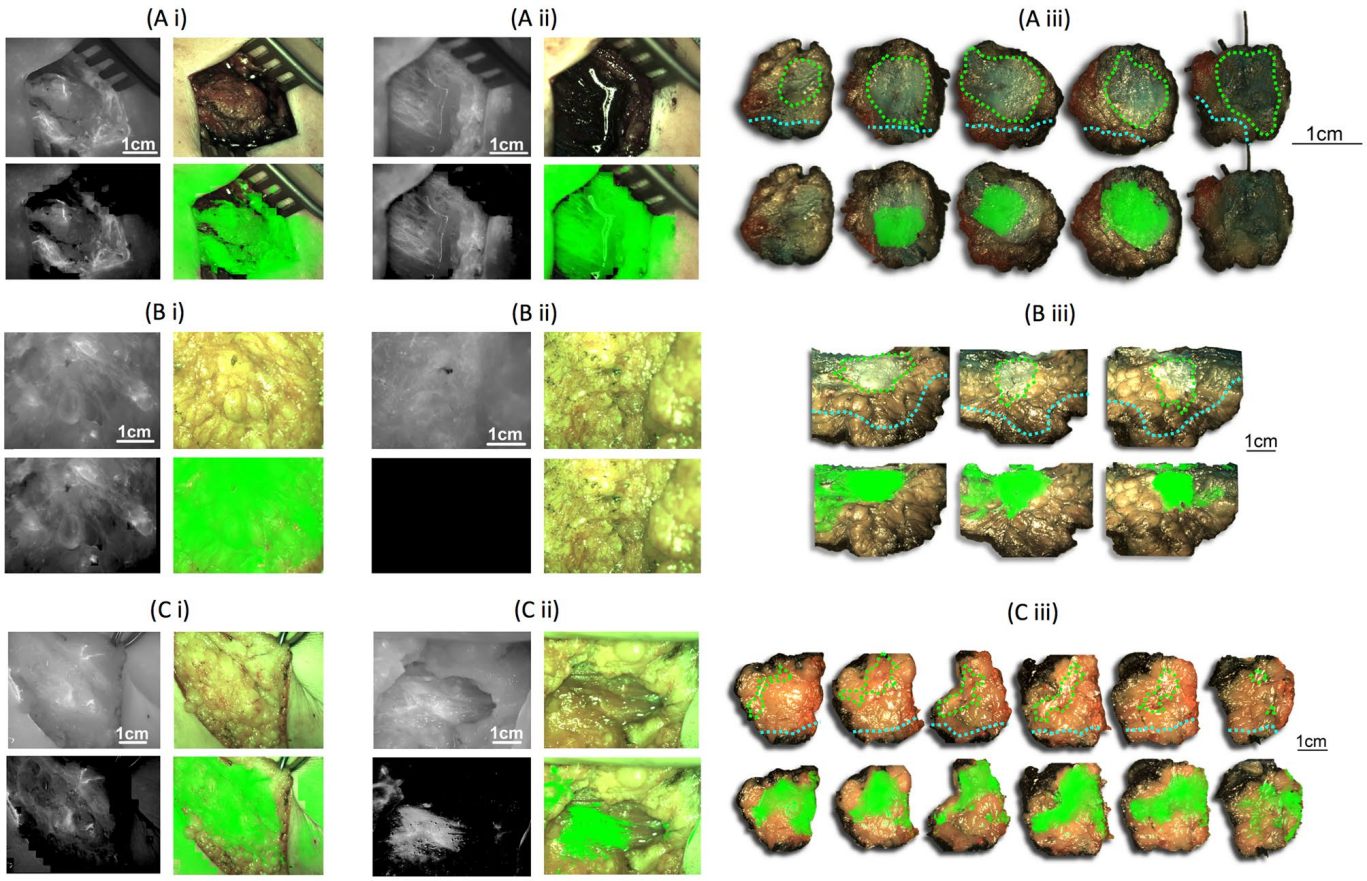
Examples of three individual clinical cases from the angiography cohort presented in three separate rows (**A**, **B**, **C**). (i) and (ii) panels demonstrate the raw fluorescence (top left) and color (top right) data and corresponding tumor probability map (bottom left) and its overlay (bottom right) for (i) the tumor *in-situ*, and (ii) tumor surgical cavity post-excision. Note that ground truth could not be marked for this data and therefore validation was not possible. Panel (iii) demonstrates the corresponding excised tumor histopathology grossing raw color image (top) with marked ground truth for tumor (green) and healthy margin (cyan) and the tumor probability map overlay (bottom). In all cases the tumor probability map was extracted with the logistic regression model, trained with both the normalized fluorescence pixel values and the slope value of the PSD curves in histopathology grossing images. Case A was found from the anterior (within 2 mm) and posterior sides (within 0.5 mm) with tumor (Sects. 1 to 5 from medial to lateral). Therefore both (Ai) and (Aii) were expected to fluoresce. Case B was found from the anterior side (within 2 mm) and from the posterior side (at > 10 mm) with tumor (10th to 12th sections in the medial to lateral direction). Therefore only (Bi) was expected to fluoresce. Case C was found from the anterior side (within 0.6 mm) and from the posterior side (within 4 mm) with tumor (Sects. 4 to 9 from medial to lateral). Therefore only (Ci) was expected to fluoresce. From panel iii it is evident that there is an agreement between the ground truth and the tumour probability map overlay apart from case A, where there are two false negative samples due to the presence of Methylene Blue whose excitation spectrum overlaps with that of ICG. Scale of each *in-vivo* image (Ai, Aii, Bi, Bii, Ci, Cii) is indicated with a bar in the raw fluorescence (top left) part. Scale of the grossing images (Aiii, Biii, Ciii) is indicated with a bar on the right side of each clinical case.

The validation of the hybrid model in the histopathology data gave the best validation scores overall in LOOCV approach (highlighted in bold, Table 2). Examples of the tumor probability map overlays in three patients after having applied the newly trained hybrid model to both tumor *in-situ* and surgical cavity images are demonstrated in Fig. 5.

### ICG LIFS study in freshly excised and formalin-fixed tissues

ICG fluorescence was detected in all samples (apart from the control). No significant autofluorescence was found on the control sample by fluorescence imaging or LIFS. The mean fluorescence peak in the freshly *ex-vivo* whole tumor specimens was at 816.9 ± 2.4 nm, while that of the histopathology grossing specimens was 812.3 ± 6.1 nm. The fluorescence peak in formalin-fixed specimens was significantly shifted by 4 nm (*p* < 0.01, Wilcoxon-Mann–Whitney test) towards a shorter wavelength.

**Figure 6:**
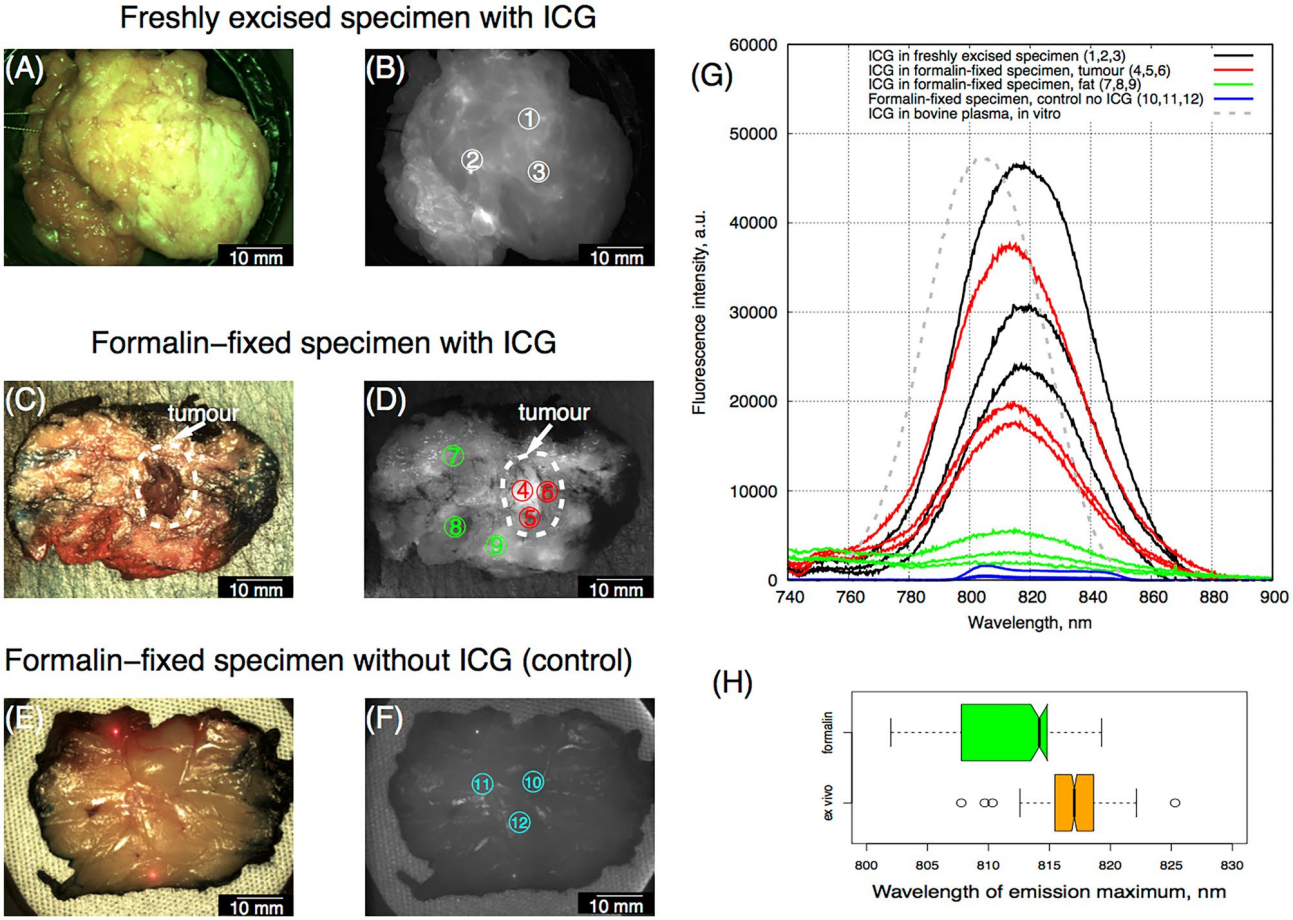
Examples of histopathology sample from formalin fixed grossed breast specimen. (**A**) Color image and (**B**) fluorescence image of a specimen from a patient (case 37) injected with 2.5 mg/kg immediately after resection (**C**) Color image and (**D**) fluorescence image of the formalin-fixed specimen from the same patient. (**E**) Color and (**F**) fluorescence image of a formalin-fixed specimen from a patient without ICG injection (control). (**G**) Examples of fluorescence spectra registered from the freshly excised specimen of the same patient (case 37) with ICG (black solid lines), from formalin-fixed tumor (red solid lines), surrounding fat tissues (green solid lines), from a formalin-fixed specimen of the control patient without ICG (blue solid lines), and the emission spectra of ICG in bovine plasma (grey dashed lines)^21^. The numbered circles indicate the approximate fluorescence spectra locations (referred to in (**G**)). (**H**) Variability of the ICG fluorescence maximum position in freshly excised specimens from four patients (orange, 563 spectra) and in the formalin fixed specimens (green, 261 spectra) is shown in (**H**). Boxes represent median, 2nd and 3rd quartile, whiskers represent maximum and minimum values.

Examples of ICG fluorescence spectra with regards to the corresponding color and fluorescence images, as well as variability of fluorescence peak position for the histopathology grossing specimens are presented in Fig. 6.

## Discussion

### Previous work and contribution

ICG injection-imaging timing has been previously studied in time domain optical mammographs *in-vivo*^14,37^ and it was found that a 25-min interval gave better sensitivity (85, 92%) but lower specificity (75, 62%) compared to imaging during ICG infusion and when ICG arterial concentration reached a steady state (sensitivity 50 versus 67%, specificity 88 versus 75%)^14^. In planar fluorescence imaging, a 24-h interval study^17^ showed good sensitivity (90% of positive margins were identified) but very poor specificity (33% of fluorescent beds post-resection had positive margins). More recently, in a study with 24-h interval in five patients, 2/5 tumors did not fluoresce at all and 1/5 tumor faintly fluoresced^38^. Finally, studies of injection at the time of anesthesia reporting sensitivity/ specificity in margin assessment of 94.2%/31.7%^39^ and 100%/60%^40^. In our study, the 25- and 5-min intervals were compared to test the hypothesis of EPR effect versus tumor hypervascularization as contrast mechanisms. A 24-h interval was not preferred to avoid potential loss of fluorescence signal^38^ and for practical logistics and surgical workflow (e.g. Monday operating lists, etc.). The chosen 25-min interval was closer to EPR timing and similar to the timing protocol in other studies^38–40^ while the 5-min interval was closer to angiography imaging as reported by Poellinger et al.^14^ These planar imaging^17,39^studies compared the overall presence of the fluorescence signal in each specimen or cavity with the corresponding positive resection margin status (as per the histopathology report) to extract specimen-based classification scores.

In the work presented here, classification (tumor vs healthy tissue) and subsequent validation was performed at pixel-level resolution. Moreover, apart from the fluorescence pixel values, the fluorescence image texture metrics were validated alone or in combination with pixel values (hybrid model) as classification model predictors. The PSD curves slope outperformed the rest of the texture metrics as a classification model predictor^21^ This was concordant with our previous findings, where the PSD metrics demonstrated superiority over the image primitives. The validation scores of the angiography cohort were found to be superior compared to the EPR cohort. This finding suggests that hypervascularization—which is a characteristic of fast growing tumours^18^—could be a better contrast mechanism than the EPR effect when fluorescence intensity (pixel values) is used for tissue classification. The half-life of ICG (5 min) results in a higher concentration within the tumor vasculature in the angiography cohort. As the EPR images were captured 25 min after the ICG injection, when the majority of ICG had been excreted, a significantly less fluorophore remained within the tumor. In addition, when texture metrics (slope of PSD curves) were used for classification, the architecture of tissue vasculature was exploited as a contrast mechanism and therefore, superiority of the angiography cohort was expected. When combining the two predictors (slope of PSD curves and pixel values), the validation scores for the grossing images (sensitivity: 0.75, specificity: 0.89) were slightly better (not statistically significant) than those found when only the pixel values were used as predictors (sensitivity: 0.82, specificity: 0.72). However, when the overlay results of whole image classification were compared (i.e. the 3rd and 5th row of Fig. 4), no substantial differences were observed. This superiority of pixel values over the PSD curves’ slope as a classification predictor may be attributed to tumor hypervascularization leading to a strong correlation between the tumor’s location and a high ICG signal, or the lower spatial frequency with which the slope of PSD curves was extracted (i.e. one slope value was extracted per 64 × 64 pixel square image sample). Moreover, in the case of histopathology grossing samples, formalin fixation may have spread the ICG in the specimen, resulting in the loss of vascular structure visualization.

Previously published work on ICG imaging of formalin-fixed breast tissue^38^ demonstrated that fluorescence, possibly of ICG, can still be detected. Our results complement this study by providing LIFS data proving that after fixation ICG remains in the tissues but also that its fluorescence spectrum is blue-shifted. According to the shape and intensity of spectra (Fig. 6), ICG was not degraded, but seems partially washed out from the tissues. An intense ICG emission peak (signal to noise ratio > 10) was found in 563 of 625 spectra (90%) acquired from freshly excised tissues, whereas in formalin-fixed samples, only 265 out of 440 spectra (59%) were identified. The fluorescence spectra emission maximum (812 nm) was shifted relatively to that of the freshly excised breast samples (817 nm). It could be theorized that this was due to the chemical changes in tissues after formalin fixation. The presence of ICG fluorescence in the formalin-fixed samples could be potentially exploited in the histopathology lab to improve or automatize preliminary breast sample analysis.

### Method validation limitations

The ground truth negative (normal tissue) was marked on a microscopic level following histopathological processing of the specimens. However, ground truth positive (tumor) could only be marked on a macroscopic level using an anterior–posterior X-ray of the specimens for the tumor *ex-vivo,* thus, any radial marking (on superior/inferior and medial/lateral views) could not be performed. For the grossed tumor, the aid of an experienced histopathologist was required to macroscopically identify the tumor. However, there was no way to determine the ground truth status for the ductal carcinoma *in-situ* (DCIS) components *and the* radial aspects of the *ex-vivo* specimen. Other tumor characteristics, affect the fluorescence signal and therefore they need to be taken into consideration when validating the technique. A bigger in size tumor would be easier identifiable, given the higher amount of present fluorophore. Furthermore, had there been a necrotic core, this would be expected to dampen the signal as the lack of vasculature would prevent the inflow of ICG for both cohorts. Based on supplementary Table S1, tumor size difference between the two cohorts was not statistically significant (*p* = 0.35) and no necrotic core was found in any of the cohorts. In the future, accurate mapping of the histopathology information onto the whole resected specimen would require a combination of macro- and micro-scale imaging and 3D reconstruction of the whole excised specimen from grossing images. Additionally, to improve estimates of the accuracy of ICG-NIR imaging for detecting residual disease on cavity walls will require further analysis of cavity shaves to obtain histopathological ground truth.

### ICG fluorescence imaging limitations

ICG emission lies within the NIR band, where there is no significant spectral overlap with autofluorescence and its signal can be detected from a surface depth up to 4 mm^41^. This depth is sufficient to establish invasive disease encroaching on a resection margin (i.e. a positive resection margin), which is considered to be disease at the inked margin as per the Society of Surgical Oncology and the American Society for Radiation Oncology (SSO-ASTRO) guidelines^42^. However, its *in-vivo* concentration, scattering and absorption variability in heterogenic breast cancer can cause inconsistencies in the fluorescence signal between images. To compensate for this, fluorescence pixel values have been normalized within each image to the maximum intensity prior to their use as classification model predictors in the LOOCV manner. However, this normalization did not compensate for spatial variations of absorption and scattering within each image while it also artificially enhanced the contrast of each image, and thus could risk false positives and negatives. To avoid this problem, fluorescence image texture metrics could be used instead of intensity as they are less dependent on the above-mentioned experimental variables. However, in the current work they were found inferior to fluorescence intensity as classification predictors. Alternatively, multispectral imaging could accompany FGS in the future to compensate for scattering and absorption albeit further system hardware modifications would be necessary to accommodate this imaging mode.

### Application of the model on *in-vivo* images

In order for this to be applied in future clinical cases, a classification model needs to be trained using previous cases’ data where the ground truth is known. To simulate this real-case scenario, the hybrid model with the best validation scores (Table 2 in bold) was trained with all the angiography cohort grossing image ground truth data and then applied to predict the tumor probability map on the *in-vivo* images (Fig. 5). Based on the margin status of the corresponding excised tumors, we speculated that the *in-vivo* application of the hybrid model gave a false positive surgical margin (Fig. 5, Ci). Four instances of fluorescent surrounding skin (Fig. 5, Ai, Aii, Ci, Cii) were also observed. The skin fluorescence could perhaps be attributed to the combination of low blood pressure secondary to anesthesia and local vasoconstriction in response to the cold exposed skin, delaying the flow through the skin (thus increasing ICG signal). Furthermore, any false positives found in the cavity during the angiography timing could be from intraoperative damage to the vasculature and subsequent bleeding into the cavity (with the blood containing ICG).

These *in-vivo* results were not taken into consideration when validating the technique, however they are in agreement with false positive observations reported by others^17,39,40^. Note that in contrast with these studies, (a) our results reflect a 5-min interval between ICG injection and imaging and (b) tissue classification and validation was implemented with pixel-level resolution. Previously reported specificity was low^17,39,40^ but our approach yielded good results (specificity of 74% in the freshly excised and 89% in the formalin-fixed tissue), thus, it could contribute towards improving tissue classification accuracy of ICG fluorescence imaging.

### Future directions

Overall, fluorescence pixel values potentially combined with PSD curve slope, have shown to be good predictors for tissue classification (tumor vs healthy). However, it is often DCIS which is responsible for positive margins. This technology is not yet capable of assessing DCIS, as adequate signal was only achieved in solid tumors visible at a macroscopic level. However, provided that the DCIS is found within range of the solid tumor, the 4 mm depth should be sufficient to achieve negative margins as per SSO-ASTRO and ABS guidelines. Furthermore, the commercially available fluorophores are non-specific, which impairs tumor detection accuracy. Therefore, the use of targeting fluorophores specific to breast cancer could further improve the accuracy^43^. However, the heterogeneity of breast cancer, with significant inter- and intra-patient variability, proves difficult to overcome. Perhaps in the future, FGS will entail using a comprehensive combination of targeting fluorophores for success. Similarly, the accompanying imaging systems would require easily customizable optical filtration which could be tailored to the fluorophore(s) of choice, combined with pixel-dense image intensity and texture-based classification as presented here, or potentially deep-learning techniques.

## Conclusion

Fluorescence imaging and LIFS studies in both freshly excised and formalin-fixed grossed specimens revealed that ICG remains in the formalin-fixed breast tissues in concentration sufficient for fluorescence imaging. Fluorescence spectra in formalin-fixed tissues are shifted towards a shorter wavelength, but the shape is not changed nor degraded. Therefore, the novel combination of ICG fluorescence intensity and texture as predictors to classify breast tissue as cancerous or healthy was validated in freshly excised tumor specimens and separately in fixed in formalin tumor grossing specimens. Validation demonstrated that these two predictors could complement each other to improve the logistic regression model sensitivity and specificity. However, fluorescence intensity, compared to texture, was found to be the dominant classification predictor. Validation of this model in formalin-fixed tumor grossing specimens yielded overall the best scores: sensitivity of 0.75 ± 0.3|specificity of 0.89 ± 0, while further model application on *in-vivo* images (tumor *in-situ* and surgical cavity) to classify tissue as cancerous or healthy was shown to be feasible. These findings encourage the use of ICG fluorescence imaging for intraoperative tumor resection guidance or as a tool to facilitate histopathology lab workflow.

## Supplementary Information

Supplementary Information 1.

Supplementary Information 2.

## Data Availability

Identifiable patient data cannot be accessed in concordance with the NHS Code of Confidentiality and the signed patient consent form. Anonymized images analyzed in the current study can only be available through the Department of Surgery and Cancer, Imperial College London and on reasonable request to the principal investigators as per IRAS protocol.
