# Supplementary material for: Indocyanine green fluorescence image processing techniques for breast cancer macroscopic demarcation
Source: Sci Rep. 2022 May 21;12:8607. doi: 10.1038/s41598-022-12504-x (PMC9124184; doi:10.1038/s41598-022-12504-x)
Supplement: Supplementary file 1 — Supplementary Information 1. [file 41598_2022_12504_MOESM1_ESM.docx]

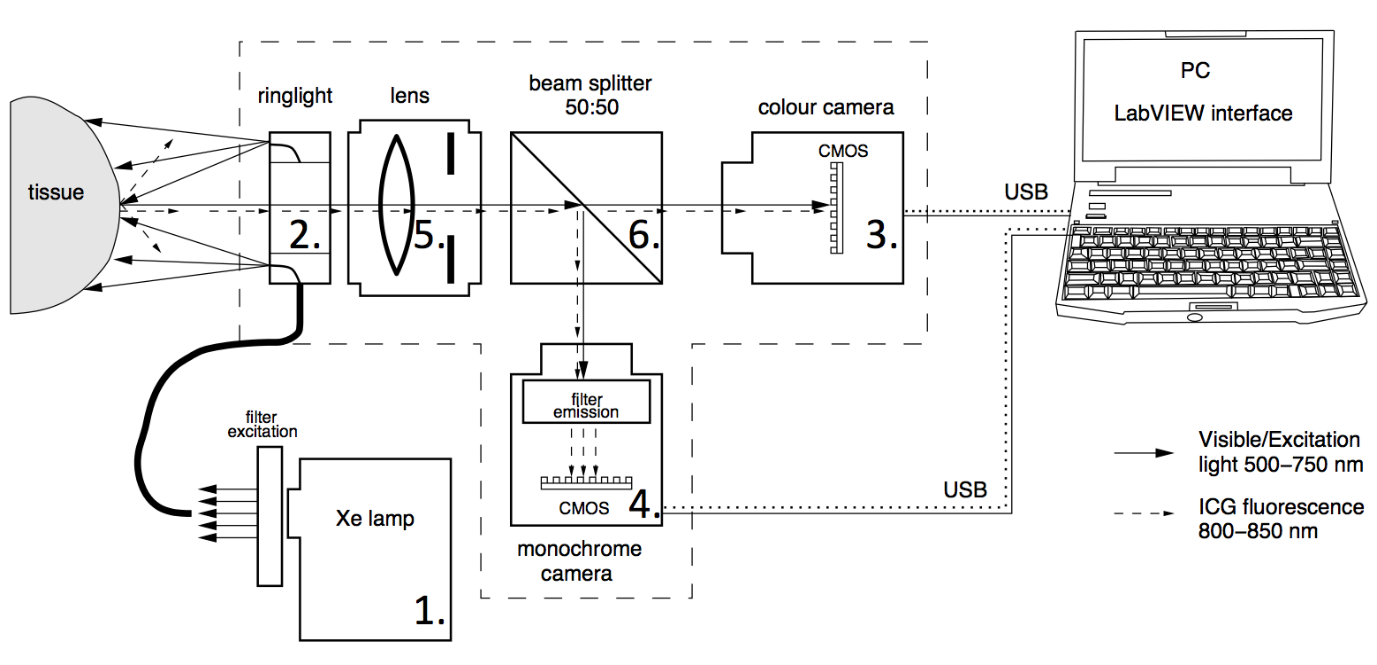


Figure S1: Schematic diagram of the in-house dual camera system. Xenon light source {1} (Max-303, Asahi Spectra Co., Ltd., Tokyo, Japan). Flexible fibre optic illumination ring {2} (Mini Annular Ringlight MA3172, Vision Light Tech B.V., Protonenlaan, Netherlands). Colour camera for visible-colour imaging {3} (FLIR Grasshoper3 GS3-U3-28S5C-C, FLIR Systems, Inc., Wilsonville, OR, USA). Monochrome camera {4} for fluorescence imaging (FLIR Grasshoper3 GS3-U3-28S5M-C, FLIR Systems, Inc., Wilsonville, OR, USA). Lens system {5} (35 mm SLR camera photographic lens, Sigma Co, Japan). 50:50 beam-splitter {6} (CCM1-BS013/M, Thorlabs, Inc., Newton, NJ, USA). The Figure was adjusted from^21^ in compliance with the Creative Commons Attribution 4.0 International License^44^.
