# Supplementary material for: Indocyanine green fluorescence image processing techniques for breast cancer macroscopic demarcation
Source: Sci Rep. 2022 May 21;12:8607. doi: 10.1038/s41598-022-12504-x (PMC9124184; doi:10.1038/s41598-022-12504-x)
Supplement: Supplementary file 2 — Supplementary Information 2. [file 41598_2022_12504_MOESM2_ESM.docx]

| **Patient Demographics** | | | | | | |
| --- | --- | --- | --- | --- | --- | --- |
|  | **EPR cohort** | | | **Angiography cohort** | | |
|  | Mean | Range (min-max age) | Standard deviation | Mean | Range (min-max age) | Standard deviation |
| Age (years), p=0.67 | 57.9 | 34-78 | ±11.7 | 56.5 | 33-81 | ±14.7 |
| BMI (kg/m2), p=0.78 | 25.63* | 20.32-36.51 | ±3.96 | 26.57* | 19.02-36.6 | ±4.89 |
| Ethnicity | % (*N*) % (*N*) | | | | | |
| White-British | 40% (8/20) | | | 10% (2/20) | | |
| White-Any other white background | 10% (2/20) | | | 15% (3/20) | | |
| Black or Black British-African | 5% (1/20) | | | 0% (0/20) | | |
| Mixed-Any other mixed background | 5% (1/20) | | | 0% (0/20) | | |
| Black or Black British-Caribbean | 0% (0/20) | | | 5% (1/20) | | |
| Asian or Asian British-Indian | 0% (0/20) | | | 5% (1/20) | | |
| Other | 40% (8/20) | | | 55% (11/20) | | |
| **Tumor Characteristics** | | | | | | |
|  | **EPR cohort** | | | **Angiography cohort** | | |
|  | Mean | Range (min-max age) | Standard deviation | Mean | Range (min-max age) | Standard deviation |
| Size (mm)**, p=0.35 | 13.0 | 1.7-30 | ±6.5 | 15.7 | 0-34 | ±9.1 |
| Histological type | % (*N*) % (*N*) | | | | | |
| IDC | 15% (3/20) | | | 15% (3/20) | | |
| IDC + DCIS | 70% (14/20) | | | 45% (9/20) | | |
| DCIS | 5% (1/20) | | | 15% (3/20) | | |
| ILC +/- ISLN | 5% (1/20) | | | 15% (3/20) | | |
| IMC + DCIS | 5% (1/20) | | | 0% (0/20) | | |
| IMPC + DCIS | 0% (0/20) | | | 5% (1/20) | | |
| FAD with atypia | 0% (0/20) | | | 5% (1/20) | | |
| Hormone receptor status |  | | | | | |
| ER+, PR+, HER2 - | 85% (17/20) | | | 70% (14/20) | | |
| ER+ (DCIS cases) | 5 %(1/20) | | | 20% (4/20) | | |
| ER-, HER2 + | 0% (0/20) | | | 5% (1/20) | | |
| Triple positive | 5% (1/20) | | | 5% (1/20) | | |
| Triple Negative | 5% (1/20) | | | 0% (0/20) | | |
| Neoadjuvant treatment |  | | | | | |
| NACT | 5% (1/20) | | | 5% (1/20) | | |
| Hormone therapy*** | 0% (0/20) | | | 5% (1/20) | | |
| Margin status |  | | | | | |
| Radial positive margins | 20% (4/20) | | | 45% (9/20) | | |
| Reoperation rate | 20% (4/20) | | | 40% (8/20) | | |

Table S1: Summary patient demographics and tumor characteristics. The table was re-created from^22^ in compliance with the Creative Commons Attribution 4.0 International License^44^. In BMI calculation two patients were excluded as height data were not available.**Mean was calculated out of only the invasive cancer cases.***In some patients where the surgery had to be delayed due to the COVID pandemic, hormonal therapy started pre-operatively.
